# Grounding the Attentional Boost Effect in Events and the Efficient Brain

**DOI:** 10.3389/fpsyg.2022.892416

**Published:** 2022-07-22

**Authors:** Khena M. Swallow, Adam W. Broitman, Elizabeth Riley, Hamid B. Turker

**Affiliations:** ^1^Department of Psychology, Cornell University, Ithaca, NY, United States; ^2^Cognitive Science Program, Cornell University, Ithaca, NY, United States

**Keywords:** predictive coding, locus coeruleus, episodic memory, temporal selection, event cognition, attentional boost effect

## Abstract

Attention and memory for everyday experiences vary over time, wherein some moments are better attended and subsequently better remembered than others. These effects have been demonstrated in naturalistic viewing tasks with complex and relatively uncontrolled stimuli, as well as in more controlled laboratory tasks with simpler stimuli. For example, in the *attentional boost effect* (ABE), participants perform two tasks at once: memorizing a series of briefly presented stimuli (e.g., pictures of outdoor scenes) for a later memory test, and responding to other concurrently presented cues that meet pre-defined criteria (e.g., participants press a button for a blue target square and do nothing for a red distractor square). However, rather than increasing dual-task interference, attending to a target cue boosts, rather than impairs, subsequent memory for concurrently presented information. In this review we describe current data on the extent and limitations of the attentional boost effect and whether it may be related to activity in the locus coeruleus neuromodulatory system. We suggest that insight into the mechanisms that produce the attentional boost effect may be found in recent advances in the locus coeruleus literature and from understanding of how the neurocognitive system handles stability and change in everyday events. We consequently propose updates to an early account of the attentional boost effect, the dual-task interaction model, to better ground it in what is currently known about event cognition and the role that the LC plays in regulating brain states.

## Introduction

Everyday experience tends to unfold predictably. Most of the time, the environment changes little from one moment to the next, and people and things behave according to learnable, predictable patterns (Saffran et al., 1996; Baldwin et al., 2008; Endress and Wood, 2011; Friend and Pace, 2011; Kidd et al., 2014; Kosie and Baldwin, 2019). However, situations can change rapidly: a task is completed, a fire alarm goes off, or a neighbor stops by with a request. In all of these cases the human cognitive system must shift from a relatively stable state that reflected the situation as it once was, to a new state that optimizes cognition and behavior in the changed environment. These aspects of everyday cognition are captured in research that examines how attention and memory dynamically respond to changes in situations and task demands. In this paper, we discuss how the *attentional boost effect* (ABE), the phenomenon whereby increasing attention to one task boosts performance in another (Lin et al., 2010; Swallow and Jiang, 2010), could reflect neurocognitive mechanisms that help people adapt to behaviorally relevant changes in ongoing events.

**Figure 1:**
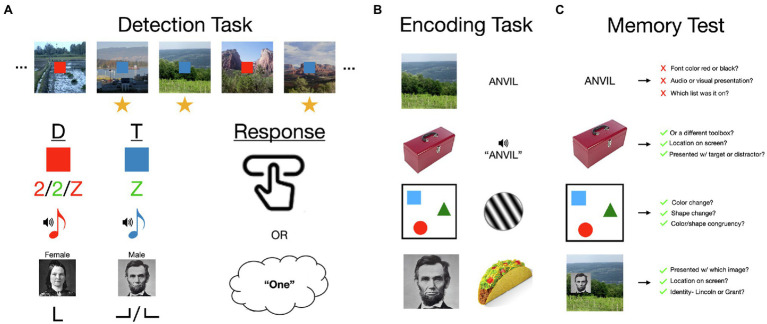
An overview of the *attentional boost effect* (ABE). **(A)** The ABE is most frequently demonstrated in a continuous dual-task for which participants memorize individually presented items (e.g., scenes; 500 ms duration; 0 ms interval) and press a button or add to a mental count when a concurrently presented but otherwise unrelated target cue is presented (e.g., a blue square target rather than a red square distractor). Memory for items presented with a target is typically significantly better than that for items paired with distractors. This paradigm has been varied considerably across studies, demonstrating several key features of the ABE: that the ABE occurs for auditory and visual detection stimuli, when targets and distractors are visually similar, and when they are defined by the conjunction of two features. The ABE can also be observed when participants respond by pressing a button, silently counting, or identifying the targets. **(B)** The ABE or similar effects have also been observed for a variety of stimuli, including short- or long-term memory for scenes, faces, objects, valuable items like food, visually and auditorily presented words, and arrays of colored shapes. Related effects occur in visual habituation to Gabor patches. **(C)** The ABE sometimes includes information that individuates an item from others that are like it, or places it within a specific momentary context. This includes information distinguishing category exemplars from each other, identifying which item appeared with which cues, and where, as well as which features were part of the same object. However, while participants may be able to remember whether a word was paired with a target or distractor, verbal materials do not otherwise appear to result in the same effects. See main text for elaboration on these findings and references to specific studies. Image attributions: Scenes reproduced from the personal library of Khena Swallow with permission, objects from https://bradylab.ucsd.edu/stimuli.html, Abraham Lincoln image is in the public domain from https://en.wikipedia.org/wiki/List_of_photographs_of_Abraham_Lincoln#/media/File:Abraham_Lincoln_O-77_matte_collodion_print.jpg, Mary Todd Lincoln image is in the public domain from https://en.wikipedia.org/wiki/Mary_Todd_Lincoln#/media/File:Mary_Todd_Lincoln_1846-1847_restored.png, button click by Schmidt Sergey from https://thenounproject.com/icon/button-click-691746/.

The ABE is difficult to reconcile with fundamental characteristics of attention—that it is limited, and therefore selective for tasks, locations, objects, and features (Kinchla, 1992; Buschman and Kastner, 2015). The standard ABE paradigm requires participants to divide attention across two tasks, for which two unrelated streams of stimuli are briefly, but simultaneously presented in a long, uninterrupted series. For the *encoding task*, participants memorize all of the images that are presented on the screen. For the *detection task* they are instructed to press a button whenever a cue has a particular feature, such as when a blue square appears (a *target*, sometimes also called *go cue*) rather than a red square (a *distractor*, sometimes also called no-*go cue*; Figure 1). The limited and selective nature of attention suggests that there should be two sources of interference in this *continuous dual-task encoding paradigm*: constant interference resulting from having to maintain two sets of goals and divide attention across two stimulus streams (Troyer and Craik, 2000; Wolfe et al., 2007) and transient redistributions of attention from the encoding task to detection task stimuli when targets occur (Duncan, 1980; Kinchla, 1992). However, this paradigm and numerous variations upon it have shown that, while dual-task interference is clearly evident in this task, transient *boosts* rather than deficits to image encoding occur when a target is detected (Swallow and Jiang, 2013). In other words, memory for images is boosted by increasing attention to an unrelated stimulus that requires a response (Mulligan and Spataro, 2014; Swallow and Jiang, 2014b).

Though the surprising nature of the ABE is rightly highlighted in the literature, it was predicted by findings in event cognition (Swallow and Jiang, 2010). The human cognitive system divides continuous experience into discrete events in a process known as *event segmentation* (Zacks et al., 2007). For example, an individual watching someone else make dinner may identify a new event (creating an *event boundary*) when the cook switches from gathering ingredients, to chopping onions, and again when the cook pulls out a pan to start sautéing the onions. Event segmentation is known to be critically involved in dynamically regulating a variety of cognitive processes, but most notably attention and memory (e.g., Newtson and Engquist, 1976; Swallow et al., 2009; Faber et al., 2018).

The ABE was proposed after it had been demonstrated that event boundaries have nearly immediate effects on the ability to recognize objects in a movie (Swallow et al., 2009, 2011). In these studies, participants watched movies that were interrupted about once a minute for a recognition memory test on an object that had been presented 5 s earlier. Event boundaries had clear effects on object recognition: objects that were visible when an event boundary occurred were remembered better than objects that never overlapped with a boundary, particularly when they had to be retrieved from a prior event. Surprisingly, these effects were present regardless of whether participants had fixated the object when it was originally presented, suggesting that encoding was broadly enhanced at these times. This research demonstrated that the *boundary advantage* in long-term memory for events (Newtson and Engquist, 1976; Lassiter and Slaw, 1991) emerged in the moment, as the event unfolded.

These and other data are explained (and were predicted) by models of naturalistic event perception that propose that the brain (and consequently the mind) is, at its core, predictive. In Event Segmentation Theory (EST; Zacks et al., 2007), event segmentation is described as a side effect of a system that minimizes computational demands by regulating memory and perceptual processing. According to EST, actively maintained representations of the current situation, called *event models*, generate predictions about perceptual input in the very near future (seconds or less; see also Zacks et al., 2011; Hasson et al., 2015; Baldassano et al., 2017; Eisenberg et al., 2018). Event models are maintained in a stable state for as long as they adequately predict perceptual information, which reduces energy demands on the system (Friston, 2009). When these predictions begin to fail, the increase in prediction error triggers a mechanism that resets the event model, causing the event to be segmented. The event model is then rebuilt using knowledge about events (e.g., knowledge about how events typically unfold) and incoming perceptual information about the current situation. As a result, representations of events should be most sensitive to perceptual information at event boundaries.

This perspective on event cognition describes how the temporal dynamics of attention and memory may reflect the transition from one stable cognitive and neural state to the next. Like increasingly prominent predictive coding views of cognition (Rao, 2005; Friston, 2009; Clark, 2013) it suggests that, to increase efficiency, the neurocognitive system forms and maintains stable states that guide ongoing perception and behavior (Richmond and Zacks, 2017). These stable states are updated only when the situation changes by temporarily increasing sensitivity to external information (Bouret and Sara, 2005). We propose that insight into the ABE, and the relationship between attention and memory more generally, may be had by directly considering whether it emerges from similar processes.

## New Perspectives on the ABE

One of the earliest challenges for research on the ABE was accounting for how increasing attention to one task (i.e., a target in a detection task) can enhance performance of another task (i.e., encoding a background item). Though this suggested that attentional capacity may briefly increase the ability to attend to external stimuli, most research on attention has been directed toward understanding how attending to one task, stimulus, feature, or modality interferes with performing other tasks or processing other information (Kinchla, 1992).

To account for the ABE, Swallow and Jiang (2013) proposed the *Dual-Task Interaction (DTI)* model. The model claims that the attentional systems that prioritize spatial locations and perceptual features operate independently of a mechanism that globally boosts attention at behaviorally relevant moments (*temporal selection*). The DTI model indicates that, in the continuous dual-task encoding paradigm, temporal selection is triggered by the decision that a detection task cue is a target and requires a response. Temporal selection was proposed to result from a phasic burst of activity in the LC, which then increased gain in the signal to noise ratio in perceptual processing. The LC is a brainstem nucleus that is the primary source of norepinephrine (NE) in the brain (Berridge and Waterhouse, 2003) and may be the major source of dopamine (DA) in the HPC (Kempadoo et al., 2016; Takeuchi et al., 2016). Phasic LC activity is most strongly associated with the decision to respond to a stimulus, preceding actions by roughly 100 ms in non-human primates (Rajkowski et al., 2004). Phasic bursts of LC activity occur in response to target detection in non-human primates and are thought to increase the contrast between signal and noise in targeted sensorimotor regions (Aston-Jones and Cohen, 2005). Because the LC projects diffusely throughout the brain (Loughlin et al., 1982; Aston-Jones and Waterhouse, 2016), the DTI model proposed that phasic bursts in LC activity could enhance the processing of all available stimuli in the environment, and these effects may be present even for information that is outside the current focus of attention.

In the nearly 10 years since the DTI model of the ABE was proposed, research has elaborated on the conditions in which the boost occurs, highlighting its generalizability and its specificity (Figure 1). At the same time, rapid advances in neuroscience and shifts in theoretical perspectives about the mind motivate new ways to think about the ABE and its relationship to event segmentation. Characterizations of LC structure and function increasingly suggest that it may have more localized effects on processing (Poe et al., 2020), implying that temporal selection could enhance memory in several, potentially independent, ways. These potential effects of the LC on memory are also increasingly grounded in perspectives that characterize the brain as active, predictive, and effort minimizing (Friston, 2009; Clark, 2013). For example, in predictive coding frameworks, the brain minimizes computational effort by generating predictions about the external state of the world that bias processing in a top-down manner. Predictions are compared to information coming in to the system, and actions re-align the system with the current state of the environment (Rao, 2005; Friston, 2009; Clark, 2013). In this framework, neuromodulatory systems like the LC may be involved in regulating the relative balance between top-down expectations and bottom-up sensory information (Clark, 2013).

**Figure 2:**
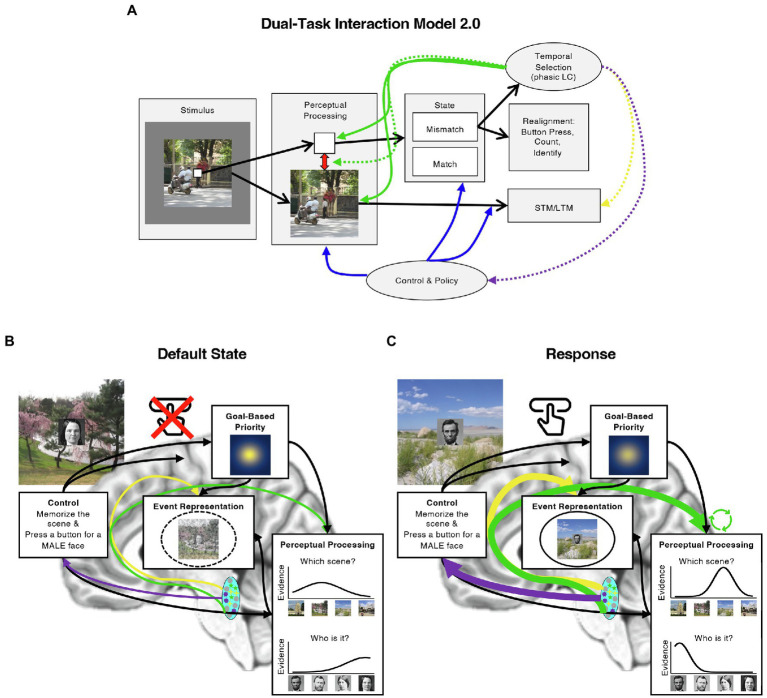
Updating the Dual-Task Interaction Model. **(A)** Illustration of the original DTI model (Swallow and Jiang, 2013) with several proposed modifications (Dual-Task Interaction Model 2.0). Like the original DTI model, we assume that presenting multiple items at once results in competition among them (red arrow in “Perceptual Processing” box; e.g., Buschman and Kastner, 2015), that control mechanisms instantiate the task by guiding attention and processing in a top-down manner (“Control and Policy” box and arrows leading from it; e.g., Badre and Nee, 2018), that temporal selection facilitates perceptual processing by increasing gain and consequently sensory precision (green arrows to “Perceptual Processing” box), and that this effect is generated by phasic LC activity. We further specify that temporal selection occurs when mismatches between the state of the world and the state of the neurocognitive system (“State” second box from the right) require a response to bring them back into alignment (“Realignment,” far right box) and that these responses may enhance the formation of bound, multi-item representations of items in the MTL (yellow dashed arrow). Computational models further suggest that phasic LC increases competition between attended and unattended items (green dashed arrow; Mather et al., 2016) or promote shifts to new neurocognitive states by briefly weakening top-down influences (purple dashed arrow; Shine et al., 2016). We note that the former possibility is based on paradigms with different timings and that there is currently little evidence to support the latter possibility. We include it for completeness. **(B)** An example of the default state of the system when the task is to both memorize scenes and press a button for male, but not female faces. Once a continuous encoding dual-task has been started, the default state of the system is to prioritize the images and faces (illustrated by the “Goal-Based Priority” box), and to withhold a button press until there is enough perceptual evidence that a male face has been presented. Perceptual processing results in image specific evidence for both faces and scenes, and these are bound to each other and their context in the MTL. **(C)** An example of the state of the system when a response must be generated. Under these conditions, we propose that phasic LC activity (blue oval) increases sensory precision in perceptual areas (green arrow; indicated by the taller, narrower evidence curve). These effects could be greater for prioritized information (green circular arrows). The result is richer sources of information for episodic encoding (“Event Representation”) in conjunction with direct enhancements to the formation of bound event memories (solid circle; it is dashed in panel **B**). It may also be possible that phasic LC activity disrupts the influence of control representations, weakening top-down biases in stimulus processing (illustrated by the weaker spotlight in the “Goal-Based Priority” box). Each of these effects could arise from LC cellular ensembles that project to different regions of the brain, regardless of whether they are independently or globally activated (illustrated with differently colored cells in the LC; see “*Modular circuitry and function in the LC*”). Image attributions: As in Figure 1, additionally, Ulysses Grant image has no restrictions from https://www.loc.gov/pictures/item/2017896375/, and Julie Grant image CC0 from https://npg.si.edu/object/npg_NPG.81.M819.

These and similar developments in the literature on the ABE and the LC prompt several elaborations on the DTI model. In the rest of this review, we therefore evaluate the DTI model in light of these new perspectives and both behavioral and neurophysiological evidence regarding the source of the ABE and its potential influences on memory. In many cases, the data motivate several updates to the original DTI model, which we present and illustrate in Figure 2 as the *Dual-Task Interaction Model 2.0*. In addition to suggesting that the ABE reflects a boost to perceptual processing, new developments in the behavioral and LC literatures suggest the possibilities that the ABE includes some aspects of episodic memory, interacts with goal-based attention, and could modulate the stability of neurocognitive states over time. We discuss each of these in turn, and describe how particular aspects of the ABE may emerge from the interaction of the LC with other systems (Figure 2B). We then describe how these developments also clarify the relationship between the ABE and event segmentation, suggesting several new avenues for investigating how attending to behaviorally relevant moments, such as when targets appear or events change, influences the uptake and encoding of information from the world.

## What Produces the ABE?

The DTI model proposed that the ABE reflects phasic LC activity, and that this activity is elicited by the decision to respond to a target cue. Relative to distractor rejection, however, target detection involves multiple processes that could be the source of the ABE. Furthermore, the nature of a “response” and how it relates to the cognitive and neural states evoked in the continuous dual-task encoding paradigm needs further elaboration.

One important possibility is that the ABE is not a boost at all. Instead, it could reflect interference from distractors rather than a boost from a target. Memory for distractor-paired items is worse than memory for items encoded under single-task conditions (Swallow and Jiang, 2010), and related phenomena have been attributed to inhibition or forgetting associated with distractor rejection (Kiss et al., 2007; Chiu and Egner, 2014). However, when a no-cue baseline condition was introduced to the dual-task encoding paradigm, memory for target-paired images was better than memory for no-cue images, although there was some evidence that distractors may interfere with memory (Leclercq and Seitz, 2012c; Swallow and Jiang, 2014b; Rossi-Arnaud et al., 2018; Meng et al., 2019). An advantage for target-paired words relative to words presented in a single task has also been found when study time is sufficiently limited (400 ms/trial; Mulligan and Spataro, 2014) and in measures of perceptual priming (Spataro et al., 2013). Thus, target detection produces a true encoding enhancement, but its effects may be washed out by processes that require sufficient time or attention to take effect.

One of the first questions to arise about the ABE was the degree to which it is driven by the contextual distinctiveness of the target (e.g., as in the von Restorff effect; Hunt, 1995). However, the ABE is easily replicated when targets and distractors are equally frequent (Makovski et al., 2011; Swallow and Jiang, 2012), though one study found that it decreases as the frequency of targets increases (Au and Cheung, 2020). The effects of target detection are also present for words that are distinct from other words presented during the encoding task (e.g., “building” in a list of animals), suggesting that they involve separable mechanisms (Smith and Mulligan, 2018). An exception is that a rare tone (occurring on 1 out of 8 trials) may boost immediate memory for coinciding scenes (Hoffing and Seitz, 2015). Thus, the evidence shows that the ABE is not simply a matter of targets “popping-out” out from distractors, though it may be modulated by the salience of the stimuli.

Because the ABE is connected to targets in these tasks, the effect could be triggered by detection task cues that partially, or completely, match the features that participants are told to search for. This is unlikely. Images paired with distractors that share features with a target are no better remembered than those paired with distractors that do not (Swallow and Jiang, 2014a). Moreover, in another study, the presence of a target was dissociated from the button press by telling participants to press a button only when there was no target on the screen. Under these conditions, memory for images that were paired with a target was impaired (Toh and Lee, 2022). This suggests that partial or full matching of items to a target stimulus is not sufficient for producing the ABE.

This research highlights, however, that another way that targets often differ from distractors is that responding to a target often involves an overt action (usually a button press). As a result, its effects have sometimes been attributed to the movement itself (Yebra et al., 2019). However, overt action is neither necessary nor sufficient for the ABE. Several studies have demonstrated that memory for items is enhanced when they coincide with a target that is covertly counted (Swallow and Jiang, 2012; Mulligan et al., 2016; Swallow et al., 2019; Toh and Lee, 2022), or whose identity is reported several seconds later (e.g., they name the white letter in a string of black letters; Lin et al., 2010). Furthermore, overt action in and of itself is not sufficient to enhance memory. One study reversed the relationship between an overt action and target detection by asking participants to press a button for all images (e.g., scenes and male faces) except when the image was in a pre-specified target category (e.g., female faces). Target faces, which required withholding a button press, were better remembered than distractor faces, which required producing one (Makovski et al., 2013). The ABE also has been found in studies that required participants to read aloud all words (Mulligan et al., 2014). In another study, increased visual cortical activity following auditory target detection was not observed following self-generated button presses (Swallow et al., 2012). Overt actions and target detection thus fail to produce the ABE and its neurophysiological correlates on their own.

The ABE thus appears to originate from the decision to respond to a cue or stimulus. We agree with Toh and Lee (2022) that the DTI model should be updated to better highlight the role of the response in the ABE rather than of a target. But, what exactly is a response? Answering this question requires addressing both when a response occurs, and what a response entails. Drawing on the event cognition and predictive coding literatures, we suggest that a response occurs when external events create a discrepancy between the current state of an organism (for example, the system may be in a “count the red letters” state) and its relationship to the world (a red letter has appeared, but it has not been counted, vs. a red letter has appeared but it does not need to be counted). This definition focuses on the relationship between the organism and the world because responses do not (and should not) occur for all types of changes in the environment (Rao, 2005; Friston, 2009; Kok et al., 2012; Clark, 2013; Baker and Levin, 2015). A stranger walking to a snack table at a crowded party may not have been predicted, but the event may also not be attended, and may be irrelevant to one’s current state. Therefore, no response would likely be generated. Rather, the mismatches that lead to response generation are those that are most relevant to understanding a situation as it relates to one’s goals (explicit or implicit), motivation, or predispositions.

Responses entail the transient mobilization of the appropriate neurocognitive, sensory, and/or effector systems to align an organism with the changed state of the world (cf. Clark, 2013; Varazzani et al., 2015; Xiang et al., 2019). The end result could be a button press (Yebra et al., 2019), incrementing an internally maintained count (Swallow and Jiang, 2012), halting repeated button presses (Makovski et al., 2013), judging how common rather than how likeable an object is (e.g., DuBrow and Davachi, 2016), or even the updating of event models (which organize perception and action) when they fail to reflect the current situation (Zacks et al., 2007; Richmond and Zacks, 2017; Baldwin and Kosie, 2020). As these examples illustrate, some responses may entail a brief mobilization of effort but without a change in how an actor interacts with the world (e.g., the task is still to count red letters), whereas others may result in larger shifts that change response contingencies (e.g., the task itself changes to judging pleasantness). We return to the potential implications of this distinction in “*How the ABE relates to the effects of event boundaries on episodic memory*.” Like earlier work, this proposal suggests that responses trigger temporal selection because they require participants to do something different than what they were doing before (Swallow and Jiang, 2012; Makovski et al., 2013; Toh and Lee, 2022). However, by emphasizing the discrepancy between an organism’s internal state and the state of the world, this definition provides a more explicit basis for thinking about the ABE in a broader cognitive and evolutionary context.

Despite defining response to include both cognitive and behavioral outcomes, it is possible that covert responses are generated by systems that, in the phylogenetic past, evolved to generate overt actions. In many invertebrates, spatially distributed neuromodulatory systems disrupt functional neural states so new states can be created and new motor behaviors generated (Katz, 1991; Bouret and Sara, 2005). In vertebrates, neuromodulatory systems carry significant anatomical and neurochemical homology to neuromodulatory systems in older life forms (Katz, 1991). Evidence from mammals, including primates (Clayton et al., 2004; Joshi and Gold, 2022), shows that activation of the LC can promote shifts from one cognitive state to another, similar to neuromodulatory-driven shifts from one motor pattern to another in crustaceans (Bouret and Sara, 2005). Indeed, many systems involved in cognitive control and attention in primates may involve motor control and planning systems (Frank et al., 2001; Anderson, 2010; Krauzlis et al., 2013).

We note that some evidence may be inconsistent with this characterization of the ABE. One study suggests that endogenously orienting attention to the moment when a target is expected to appear may enhance memory to a comparable degree as responding to a target (Sisk and Jiang, 2020). Hoffing and Seitz (2015) also found better memory for scenes paired with an oddball tone. Furthermore, though rewarding participants for their rapid responses to targets does not appear to increase the magnitude of the ABE (Yebra et al., 2019), other findings suggests that the effect of target detection on memory may be weaker for arousing, aversive, or unusual stimuli (Mulligan et al., 2014; Spataro et al., 2014; Rossi-Arnaud et al., 2018; Yebra et al., 2019), implying overlapping sources. We suggest that some of these events may be stronger indicators than others of a mismatch between the current state of the system and the state of the world, and that a response of some sort may be needed, even if that response is not defined by the task. Indeed, salient, unexpected stimuli have long been linked to the phasic activation of the LC system and increased flexibility in cognitive state (Bouret and Sara, 2005). Careful consideration of this and other possibilities in future research will help further characterize the relationship between variations in attention over time and the encoding of information from the environment.

Finally, the original DTI model also suggested that temporal selection may be more strongly enabled by the regular onset of trials in the continuous dual-task encoding paradigm (Swallow and Jiang, 2013). Attentional selection may be more efficient if it can capitalize on the rhythmic structure of experience (e.g., in eye movements; Schroeder et al., 2010; Besle et al., 2011; Zion Golumbic et al., 2013). Oscillatory activity in the brain also appears to play an important role in regulating the updating of information over time and space generally, and of phasic LC activity specifically (Buschman and Kastner, 2015; Sara, 2015; Totah et al., 2018). However, direct evidence that the ABE is stronger with rhythmic processing has yet to be published, and the findings that led to this proposal (Makovski et al., 2011; Swallow et al., 2012) may be explained by the longer duration of trials in these paradigms (Mulligan and Spataro, 2014). Additional research is therefore needed to characterize the relationship between temporal selection and oscillatory brain activity.

### Evidence That the ABE Is Tied to Phasic LC Activity

The proposal that the ABE reflects phasic LC activity (Swallow and Jiang, 2010) stemmed from the contemporaneous view that afferent projections from the LC contacted nearly every part of the brain (with the exception of the striatum), including perceptual areas, and appeared to be relatively undifferentiated in their behavior (Aston-Jones and Cohen, 2005). This pattern, in conjunction with the clear relationship between phasic LC firing and decisions to respond to targets (Rajkowski et al., 2004; Aston-Jones and Cohen, 2005), suggested that the LC system could reasonably support the temporally precise, but spatially diffuse, effects that were hypothesized to produce the ABE. In this section we review evidence for whether the ABE is related to phasic LC activity.

Though there has been a burgeoning interest in characterizing the cognitive mechanisms of the ABE and its impact on memory, only a handful of studies bear on its neurophysiological basis. Most of these have used an indirect measure of LC activity, pupil size, which increases with activity in the LC (Murphy et al., 2014; Joshi et al., 2016). Hoffing and Seitz (2015) found increased pupillary responses and better immediate recognition memory for images that were paired either with an alphanumeric target (e.g., a letter), or with an unexpected sound, than for other images. Swallow et al. (2019) came to a similar conclusion with covert counting of auditory targets and an extended encoding period before a final recognition memory test. Larger pupillary responses to targets than distractors only occurred when the scene was subsequently remembered rather than forgotten. Similar results were reported by Yebra et al. (2019) who found that encoding related pupil dilation was greatest when a subsequently remembered object was paired with a cue to press a button.

Linking the ABE to phasic increases in pupil diameter supports its association with phasic LC activity, but is not sufficient. Though LC activity drives changes in pupil size, pupil size ultimately reflects the combined effects of several neuromodulatory systems and subcortical structures (Larsen and Waters, 2018), is correlated with activity throughout the brainstem (de Gee et al., 2017), and can produce correlation maps that differ from those produced by LC activity (Turker et al., 2021). Furthermore, studies examining the relationship between the ABE and cardiovascular activity, which suppresses LC activity during the systole phase of the cardiac cycle, have provided mixed results (Li et al., 2018, 2020).

More direct measures of activity in the LC are needed to evaluate the hypothesis that the LC is involved in generating the ABE. This can be found to some extent in fMRI studies examining the effects of target detection on BOLD activity. In one study, auditory target tones, but not distractor tones or no tones, increased BOLD activity in LC regions defined using neuromelanin imaging (Moyal et al., in press). These conditions also increased BOLD activity in early visual processing regions (Swallow et al., 2012; Moyal et al., in press). Ventromedial prefrontal cortex may also be preferentially activated by target-paired images (Schonberg et al., 2014). Yebra et al. (2019) further demonstrated an interaction between cue (target vs. distractor) and subsequent memory in activation of brainstem voxels consistent with the LC. These effects are consistent with known LC projections and support the idea that LC activity, when engaged, makes the difference between a stimulus being remembered and a stimulus being forgotten. This conclusion is broadly consistent with findings that schizophrenia, bipolar disorder, and aging, which can sometimes negatively impact LC function, are associated with a reduction in the magnitude of the ABE (Rossi-Arnaud et al., 2014; Bechi Gabrielli et al., 2018, 2021; Prull, 2019).

Notably, there are characteristics of LC activity that have not yet been investigated in the ABE, or for which there is little evidence that they play a role in the effect. One major point of disconnect is that phasic LC responses are larger when targets are less frequent (Berridge and Waterhouse, 2003). Few studies have directly compared the impact of rare versus frequent targets on the magnitude of the ABE. However, two studies found comparable ABEs in both short-term and long-term memory tasks for rare and frequent targets (Makovski et al., 2011; Swallow and Jiang, 2012), while a third study suggested that frequent targets produce a smaller boost in long-term memory (Au and Cheung, 2020). Computational modeling suggests that phasic LC responses to Go cues should occur even when they are slightly less frequent than No-Go cues (Sales et al., 2019), so it is possible that stronger manipulations are needed to consistently observe these effects in the ABE. Another, related characteristic of phasic LC activity is that it habituates over time (Berridge and Waterhouse, 2003). There is relatively little research on habituation in the ABE paradigm. However, one study did not find a reduction in the encoding boost when a target was preceded by several target cues rather than distractor cues (Yebra et al., 2019). Finally, the timing of the effects of phasic LC activity on how information is processed and encoded into memory is far from clear. Non-human animal research suggests that phasic LC activity could influence processing as much as 300 ms after the onset of a stimulus (Devilbiss and Waterhouse, 2011). However, images that appear 100 ms after a target are not better remembered than those that appear immediately after a distractor (Swallow and Jiang, 2011), unless the cue remains on the screen when the image is presented (Yebra et al., 2019). These are important characteristics of phasic LC activity that should be examined in future research.

Though we have focused on the LC, many characteristics of LC function overlap with those of the DA, serotonergic, and cholinergic systems, and all of these may interact with each other (Briand et al., 2007). For instance, detection of Go cues in Go/No-Go paradigms produces brain-wide DA neuromodulation (e.g., Guitart-Masip et al., 2012), which impacts attention (Niv et al., 2015). Serotonin may also play an enabling role in visuo-spatial processing (Park et al., 1994). Like NE, acetylcholine enhances attentional precision and transiently biases HPC dynamics to boost encoding (Hasselmo and Sarter, 2011; Decker and Duncan, 2020). Nevertheless, there are also important differences between neuromodulators. For example, acetylcholine may aid in the maintenance of a given brain state whereas NE may not (Munn et al., 2021). Thus, although we currently consider phasic LC activity to be the most promising candidate for generating the ABE, other systems may also play a role. Future research is needed to tease apart the influence of each system.

### How Might Responses Boost Memory in the ABE?

Though research on the ABE suggests that responses are necessary to produce it, it is unclear how responses boost memory. The DTI model suggests that the cognitive mechanism responsible for the ABE is *temporal selection*, which prioritizes the perceptual processing of information that is encountered at a specific time. It also proposes that temporal selection reflects the phasic release of NE from the LC. Because the ABE is most frequently examined with memory measures, however, it could reflect effects on a variety of perceptual and cognitive processes that intervene between the presentation of an item and performance on the memory test. These possibilities are explored in the next sections.

As will become evident, the proposal that the ABE results mainly from a boost to perceptual processing does not clearly address several new findings from the literature. However, current research describes several ways that phasic LC activity could influence memory encoding. These include the potential role of the LC in (1) modulation of perceptual regions, boosting bottom-up signal from the external environment, (2) modulation of HPC sensitivity to new environmental information, promoting the formation of new mental models of the external environment or events in memory, and (3) further enhancing the effects of goal-directed attention and salience on competitive interactions in perceptual processing. The effects of LC activity on the ability to shift to new cognitive states may also provide additional insight into the relationship between the ABE and event segmentation. We discuss each of these possibilities and their relationship to extant data in the following sections. We emphasize, however, that these mechanisms are not necessarily mutually exclusive and their effects may be independent of or in conjunction with others.

## Perceptual Processing May be Boosted in the ABE

Research on the ABE provides broad consensus on two aspects of what it captures: that its effects generalize to information presented across modalities and materials, and that its effects on encoding occur, at the least, early on. The ABE has been generalized to a broad spectrum of encoding materials, including arrays of colored shapes, objects, faces, and visually and auditorily presented words (e.g., Mulligan et al., 2014; Li et al., 2018; Sisk and Lee, 2021; Spataro et al., 2021; Figure 1B) However, it does not appear for all types of stimuli equally. For example, in a series of studies examining the ABE for words, Mulligan and colleagues showed that the ABE is stronger for high frequency words than low frequency words (Mulligan et al., 2014; Smith and Mulligan, 2018) and is reduced for orthographically distinctive words (Spataro et al., 2014), which are thought to attract attention early in encoding. The ABE therefore appears to influence memory for a wide range of stimuli, but may have larger effects on items that would otherwise be more poorly remembered.

Still other evidence suggests that the ABE enhances early encoding mechanisms. The ABE is present only for displays that overlap with a target in time: it is not observed when the target appears immediately before an image, immediately after an image, during the retention interval, or during retrieval (Makovski et al., 2011; Swallow and Jiang, 2011). The ABE also appears to be stronger when trials are shorter and elaborative processing is limited. In a particularly informative study with verbal materials, Mulligan et al. (2014) found that the magnitude of the ABE decreased as trial duration (and consequently encoding time) increased. Increasing encoding time resulted in greater gains for distractor-paired words than for target-paired words, suggesting that whatever generates the ABE, its benefit can be offset by later processes. This finding is consistent with an effect of temporal selection on early encoding processes. However, limiting the opportunity to encode an item too much may reduce or eliminate the ABE with visual materials: the ABE is weak when encoding time is limited to 250 ms (Hutmacher and Kuhbandner, 2020).

Evidence for early effects on encoding also can be found in tasks that are sensitive to perceptual processing. The ABE has been observed in implicit measures of perceptual priming but not in measures of conceptual priming (Spataro et al., 2013, 2017). The ABE also incorporates enough perceptual information to allow people to better distinguish scenes from their mirror-reversed counterparts (Swallow and Jiang, 2010) and exemplars within the same category of objects (Sisk and Lee, 2021) or faces (Turker and Swallow, 2019). However, these effects may not generalize to the color, font, or modality of verbal materials (Mulligan et al., 2016). Target detection also enhances visual short-term memory for faces, arrays of 3 or 5 colored squares, and combinations of shape and color (Makovski et al., 2011; Li et al., 2018; Spataro et al., 2020). Psychophysical studies are also consistent with an effect on perceptual processing. Perceptual learning of sub-threshold visual features is enhanced following their repeated pairing with targets in an unrelated detection task (*task-irrelevant perceptual learning*; Seitz and Watanabe, 2009). Target detection has also been shown to increase habituation to tilted gratings (Pascucci and Turatto, 2013). Finally, detecting a target (e.g., an auditory tone) increases BOLD activity in perceptual regions of the brain that would not normally be involved in processing it (e.g., primary visual cortex; Swallow et al., 2012; Moyal et al., in press).

In summary, consistent with the DTI model, a wide range of data and measures suggest that the condition that generates the ABE—responding to a cue—influences perceptual processing. As a result, its effects extend to a wide variety of materials, across modalities, and emerges in multiple measures. This is true as long as later processing is sufficiently limited by dual-task interference or brief trial durations and the items are not inherently likely to attract attention early in encoding (e.g., Smith and Mulligan, 2018).

### Increasing Sensory Precision by Boosting Gain in Perceptual Areas

Like the original DTI model we suggest that the early effects of responses on perceptual processing reflect the phasic release of NE in perceptual processing regions of the brain (Swallow and Jiang, 2013; green lines in Figure 2). In this way, the phasic release of NE may increase the influence of sensory information on higher level processing, allowing it to more strongly influence representations that capture the state of the world (e.g., event models).

This proposal is consistent with a large number of findings on the impact of LC on sensory processing (Waterhouse and Navarra, 2019). When background LC activity is moderate, NE increases the excitability of sensory neurons and suppresses spontaneous discharge, resulting in an increase in the signal to noise ratio, or sensory gain, in these regions (Berridge and Waterhouse, 2003; Aston-Jones and Cohen, 2005; Devilbiss, 2019). LC activity has also been shown to sharpen the receptive fields of sensory neurons and increase functional connectivity between thalamic and cortical sensory neurons (Hurley et al., 2004; Devilbiss, 2019). In one study using optogenetics, phasic activation of the LC resulted in sensory neurons exhibiting enhanced responses to sensory input, comparable to the effects of increasing stimulus intensity (Vazey et al., 2018). Human neuroimaging data also suggest that NE modulates sensory gain and enhances sensory precision, or the amount of information that can be decoded from neural activity (Eldar et al., 2013; Warren et al., 2016). However, it should be noted that both studies were more focused on the impact NE availability over extended, rather than brief, periods of time. This literature suggests that the phasic activation of the LC following the decision to respond to a cue during an encoding task could increase sensory precision. Consistent with this possibility, responding to auditory targets boosts LC activity and the amount of decodable information in patterns of activity in ventral visual cortex (Moyal et al., in press). As a consequence, systems involved in encoding these moments into memory may have a richer, more detailed perceptual representation with which to work.

However, relatively few studies have directly examined the effects of temporal selection on perceptual processing, or its relationship to phasic LC activity. Future research should further investigate the impact of responses on the momentary availability of perceptual information for encoding using more proximate and sensitive measures of visual and auditory processing. Characterizing how responses and context changes modulate the quality and quantity of information processing in psychophysical studies, or how they impact decision thresholds and evidence accumulation rates in perceptual decision making would also be informative.

## Temporal Selection May Also Directly Enhance Episodic Memory

If the ABE is related to the effects of boundaries on event memory, then it may capture information about the event in which an item was encountered, not just the item itself (Rubin and Umanath, 2015; Moscovitch et al., 2016). There are several aspects of encoding that contribute to episodic memory, including those that bind features of objects (Erez et al., 2016), that bind items to their spatial or temporal context in episodic memory (Eichenbaum, 2004; Hannula et al., 2007), or that prioritize valuable items for subsequent memory (Shohamy and Adcock, 2010). The available data suggest that some, but not all, of these mechanisms could be at play in the ABE.

Responding to targets may facilitate the binding of object features, like color and shape, into object representations. Participants are better able to report a task-irrelevant feature of a target than a distractor (e.g., the shape of the cue when color defines whether the cue is a target or distractor; Turker and Swallow, 2019). A short-term memory study (Spataro et al., 2020) found responses during encoding may improve participants’ ability to report when two items on the screen swapped colors, a condition that may reflect feature binding (cf. Wheeler and Treisman, 2002). These effects seem unlikely to be limited to short-term memory, as responding to targets also enhances the ability to distinguish within category exemplars (Sisk and Lee, 2021). This suggests that responses can facilitate the binding of features of visual stimuli into a unified representation that individuates them from others, an ability that contributes to episodic memory (Erez et al., 2016).

One study suggests that responses do not enhance memory for the perceptual features of words (Mulligan et al., 2016). However, visual materials may produce different results (cf. Intraub and Nicklos, 1985; Weldon et al., 1995; Onyper et al., 2010; Baddeley and Hitch, 2017) perhaps because participants may prioritize different types of information when memorizing visual rather than verbal materials. This explanation implies that the ABE interacts with goal-directed attention, a possibility that we discuss in “*Goal-directed attention modulates the effects of temporal selection on encoding*.” Additional research is needed to understand the discrepancy between the effects reported with words and with visual stimuli, and to ensure that findings demonstrated with one type of stimulus generalize to the other.

Episodic memory includes the ability to bind items to their locations or to other items on the screen (Konkel and Cohen, 2009; Ranganath, 2010). There is mixed evidence that this type of binding is enhanced in the ABE. Several studies have demonstrated that participants are more likely to report that an item was paired with a target during encoding when it actually was presented with a target rather than a distractor (Swallow and Atir, 2018; Turker and Swallow, 2019; Mulligan et al., 2021). However, this specific effect could reflect a strength-based inference that better remembered items were more likely to have been paired with a target rather than a distractor cue (Mulligan et al., 2021). Other evidence cannot be attributed to such an inference. In these studies, a target-related advantage was found when participants were asked to distinguish between two options that were unrelated to whether the image was presented with a target or distractor: on which side of the screen a scene appeared (Leclercq et al., 2014), where the detection task cue appeared relative to the scene, and the identity of the cue itself (Turker and Swallow, 2019). These findings align with work showing that participants’ subjective ratings of memory quality are enhanced for target paired items (Leclercq et al., 2014; Meng et al., 2019; Yebra et al., 2019; Broitman and Swallow, 2020). Together, these results imply that the ABE can sometimes incorporate the momentary context in which an item appeared.

In contrast, it appears unlikely that temporal selection enhances episodic memory through other means. Current evidence argues against responses enhancing the formation of inter-item associations (Mulligan et al., 2016; Spataro et al., 2021) or access to semantic associations (Spataro et al., 2017), at least with verbal materials. Thus, any effect of responses on binding items to their context may be limited to information presented in that moment. We note, however, that whether target detection boosts temporal context memory for visual materials has not been adequately tested and there is some evidence that it could. Several studies demonstrating an ABE did so with a task that required participants to know not just that a scene had been presented, but whether it was presented on the previous trial (Lin et al., 2010; Leclercq and Seitz, 2012b; Hoffing and Seitz, 2015). The ABE in these tasks could thus reflect better memory for when an image was presented.

Another possibility explored in the literature is whether the ABE enhances memory by modifying the perceived value of items that appear with targets relative to distractors. Pairing an image with a target cue rather than a distractor cue increases its perceived value (*cued approach*; Schonberg et al., 2014), leading to a willingness to pay more for it, greater liking and trust ratings for faces, and greater wanting ratings for objects (Swallow and Atir, 2018; Li et al., 2020; Botvinik-Nezer et al., 2021). This raises the possibility that the ABE is due to changes in the perceived value of target-paired items (cf. Shohamy and Adcock, 2010). However, the evidence indicates that value does not improve image memory in the standard ABE paradigm (Swallow and Atir, 2018) and that the effect of responses on perceived value reflects better memory for these items, rather than the reverse (Botvinik-Nezer et al., 2021).

The behavioral data thus suggest two possibilities: (1) consistent with the DTI model, temporal selection boosts perceptual processing only, resulting in a richer source of information for subsequent binding and episodic memory formation mechanisms to act on; and (2) temporal selection additionally facilitates the formation of bound, multi-item representations in memory. Neural data provide some support for the latter possibility: to the extent that the HPC and broader MTL support the formation of bound episodic representations, findings that responding to a target increases connectivity between the parahippocampal gyrus and putative LC (Yebra et al., 2019), and between the HPC and visual cortex (Moyal et al., in press) provide evidence that its impact is not limited to early visual or auditory cortical activity. If this is the case, then the original DTI model may be incomplete.

### Direct Modulation of Episodic Encoding in Medial Temporal Lobe

The original DTI model suggests that temporal selection enhances memory encoding by improving or speeding perceptual processing. However, observations that temporal selection enhances key characteristics of episodic memory points to the involvement of processes that individuate remembered items and events, tying together what was present and where. Because this ability is critically dependent on the HPC and MTL (Moscovitch et al., 2016) we propose that, in addition to boosting perceptual processing, temporal selection may directly enhance encoding in the MTL (yellow line in Figure 2). Like event models, the resulting representations may then contribute to internal characterizations of the current state of the world and how one may act within it.

The involvement of phasic LC activity in episodic memory formation has received growing support in the non-human animal and neuroimaging literatures. The LC projects directly to the HPC, and may be the primary source of DA and NE in the dorsal hippocampus in rodents (Kempadoo et al., 2016; Takeuchi et al., 2016; Seo et al., 2021). LC activity enables the recognition of novel environments (Grella et al., 2019) and learning new contexts and spatial layouts (Kempadoo et al., 2016; Takeuchi et al., 2016; Wagatsuma et al., 2018). Phasic LC activity has also been tied to shifts in HPC representations, allowing animals to associate learning periods with separate episodes in memory (Grella et al., 2019). Activation of LC neurons appears to enhance stimulus related activity in the HPC tens of milliseconds later (Quinlan et al., 2019) and impact theta and gamma oscillatory activity in CA1 subfield (Sara, 2015). These results have been interpreted as implying an attentional role of phasic LC activity in HPC mediated associative memory (Kempadoo et al., 2016; Quinlan et al., 2019).

There is also growing evidence that the LC modulates hippocampal processing in humans. In addition to findings that responses to cues may increase connectivity between the MTL, the LC and visual cortex (Yebra et al., 2019; Moyal et al., in press), LC activity is correlated with activity in the HPC during rest (Jacobs et al., 2015; Turker et al., 2021). Degradation in the LC has been further linked to cognitive impairments in dementia (Jacobs et al., 2015; Giorgi et al., 2017) and individual variability in a range of memory performance measures in older adults (Mather et al., 2016; Lee et al., 2018; Dahl et al., 2019). These findings may provide some insight into the reduced ABE in older adults (Bechi Gabrielli et al., 2018; Prull, 2019). LC-NE release may also increase the likelihood that an old object will be perceived as new and increases the ability of the HPC to distinguish similar stimuli and contexts from each other (i.e., pattern separation; Yassa and Stark, 2011; Segal et al., 2012; Jefferies and Di Lollo, 2018).

These data point to a critical role of the LC in memory encoding and consolidation in the MTL. However, there are many differences between the paradigms in which LC contributions to HPC dependent memory have been shown in non-human animals, and those involved in the ABE. One important question is whether LC modulates episodic memory for smaller shifts in task demands, like the presentation of a cue requiring a response, or only occurs for more salient changes, like entering a novel environment or encountering an aversive stimulus. Another concern is whether temporal selection during encoding results in better consolidation of items presented with, before, or after the cue to respond. Many (but not all, cf. Mather et al., 2016; Grella et al., 2019; Quinlan et al., 2019) of the studies examining LC’s contributions to episodic memory focus on its role in memory consolidation, which is enhanced for information presented prior to emotionally arousing events (Anderson et al., 2006; Mather et al., 2016). Investigations directly examining the effects of temporal selection on representations in the MTL, as well as its influence on pattern separation and pattern completion are needed. Furthermore, examinations of how the ABE changes with more salient cues and delay intervals of 24 h or more could better characterize its relationship to arousal and memory consolidation.

## Goal-Directed Attention Modulates the Effects of Temporal Selection on Encoding

The original DTI model proposes that temporal selection operates independently of, or along-side, mechanisms that prioritize locations and features in a scene. This proposal stemmed from the observation that attending to a target in a detection task resulted in better memory for other unrelated information presented at that time. However, it also implies that, like the boundary advantage (Swallow et al., 2009, 2011), the ABE could incorporate information that is outside the current focus of attention. The results of studies investigating this issue have been mixed: some studies report a boost to ignored or task-irrelevant information (Dewald et al., 2013; Swallow and Jiang, 2014a; Walker et al., 2017; Turker and Swallow, 2019; Yebra et al., 2019; Broitman and Swallow, 2020), others report no effect of target detection on memory for ignored images (Swallow and Jiang, 2011; Leclercq and Seitz, 2012a; Hutmacher and Kuhbandner, 2020), and one study found poorer memory for ignored background words paired with targets (Dewald et al., 2011).

One factor that may account for the variety of effects of target detection on incidental memory is whether the images could be attended despite instructions to ignore them. As argued by Hutmacher and Kuhbandner (2020), the presence of an ABE for incidentally presented images may depend on how long, and how many times, they are presented. When participants were instructed to memorize the objects presented in 250 ms long trials, they showed a small memory advantage (~2%) for objects that appeared with a target. No effect was observed when the objects were ignored. The authors suggested that participants may attend to irrelevant items that are presented multiple times or for longer durations. Consistent with this argument, those studies that have shown an ABE for incidentally encoded images have typically presented them more times or with longer trials than those that have not.

However, the small magnitude of the ABE with 250 ms long trial durations raises another possibility: that the ABE depends on having sufficient opportunity to encode the images, even when they are intentionally memorized. If the mechanisms that generate the ABE are engaged by the response (Toh and Lee, 2022), then presenting a new image every 250 ms could disrupt the effect. Indeed, the ABE may be modulated by the opportunity to encode the images even when participants are instructed to memorize them. In a series of experiments, Broitman and Swallow (2020) found that a response-related boost to recollection, but not familiarity, was comparable for intentional and incidental encoding instructions. However, for intentionally encoded faces the recollection effect was present only when participants had sufficient time (2000 ms total) to study the face, whether within a single study trial, or spread out across two. Others also have reported greater recollection, but not familiarity, rates in surprise memory tests of target-paired objects presented for 1 s (Yebra et al., 2019). These findings are consistent with research suggesting that visual memory for complex scenes can require longer than 250 ms to be established even when tested immediately (Liu and Jiang, 2005), and that context memory may require sufficient encoding opportunity to manifest (Malmberg and Nelson, 2003; Litman and Davachi, 2008).

Whether the ABE can incorporate information that is outside the current focus of attention is therefore unresolved. We believe, however, that the bulk of the evidence supports two conclusions for now. First, target detection does not reliably increase dual-task interference for ignored background images. This was true even when ignored images were presented very briefly and/or only one time (e.g., Yebra et al., 2019; Broitman and Swallow, 2020; Hutmacher and Kuhbandner, 2020). Second, at the least, goal-directed attention to the background item increases the magnitude of the ABE (Swallow and Jiang, 2014a; Broitman and Swallow, 2020). The effect of target detection on memory for background items may be difficult to detect when participants are given a surprise memory test on the items. But it is highly replicable when participants are told of the memory test in advance. It is unclear how these effects can be explained by the DTI model in its original formulation. In the next section we describe a mechanism that could produce such an effect.

### Interactions Between Stimulus Priority and NE in Perceptual Processing

The observation that the magnitude of the ABE is itself boosted for attended or intentionally encoded information suggests that the effects of phasic LC activity on sensory precision or episodic encoding may interact with those of goal-directed attention (green dashed line in Figure 2). One prominent model of LC function describes a process by which such effects could emerge. In the Glutamate Amplifies Noradrenergic Effects (GANE; Mather et al., 2016) model, phasic arousal further boosts the processing advantage of items that have been prioritized and inhibits those that have not *via* local, positive feedback loops. According to this account, cortical areas representing prioritized information are expected to be high in glutamate, which promotes the local release of NE. NE then amplifies competitive interactions between more active and less active representations through local inhibition, and promotes more release of glutamate in the prioritized areas, which further increases NE release. These dynamics could help explain why the ABE is easier to detect for background scenes when they are intentionally memorized. Under these circumstances, control and frontoparietal systems may prioritize the scenes relative to other sources of information during task performance (e.g., the room in which the task occurs) throughout the task, only to have these effects magnified by positive feedback loops between glutamate and NE when the LC is physically activated by the decision to respond to a target.

The GANE model may provide a basis for thinking about how temporal selection could interact with the prioritization of different sources of information by attention. However, there are reasons to be cautious about its ability to account for the effects of goal-directed attention in the ABE. It is unclear why responding to a target does not more consistently interfere with incidental memory for background items, especially under conditions that limit their processing. To our knowledge only one study has found that unattended background items are more poorly remembered when they are paired with a cue that required a response (Dewald and Sinnet, 2012). Furthermore, the GANE model focused on interactions between arousal induced by aversive events (the presentation of a conditioned stimulus associated with an electrical shock) and visual salience or attentional priority of subsequently presented stimuli (e.g., Lee et al., 2018). It also describes effects that may evolve over longer time intervals than those in the ABE (e.g., Sakaki et al., 2014). Additional research further clarifying the time course of the ABE and its relationship to arousal induced by aversive events should provide additional insight into whether the GANE model helps to explain how the ABE is modulated by goal-directed attention.

## How the ABE Relates to the Effects of Event Boundaries on Episodic Memory

**Figure 3:**
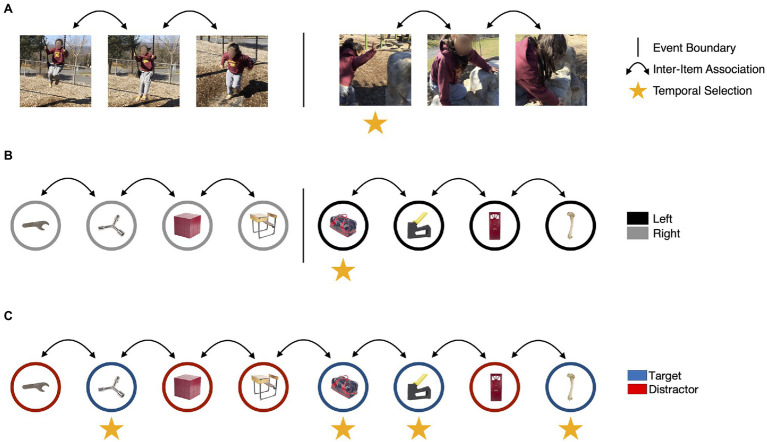
The ABE may capture some, but not all, of the mechanisms that guide attention and memory for everyday events. **(A)** During naturalistic experiences, changes in location, goals, or behavior may cause events to be segmented (Zacks et al., 2010), as indicated by the vertical bar. Event segmentation may disrupt context representations and lead to the establishment of new event models to guide cognition and behavior. This process could reduce the accessibility of perceptual and conceptual features of items encountered before the change (i.e., the color of the swing) and enhance the encoding of items that were on the screen during an event boundary, as indicated by the vertical arrows. **(B)** In laboratory studies, event boundaries may be evoked by changing task instructions during an encoding task. In this task modeled on Clewett et al. (2020), a tone is played into one ear each time an object is presented. If the tone is played in the left ear, participants respond to a judgement task (e.g., is this object more likely to be found inside or outside?) using their left hand. If the tone is played in the right ear, participants respond with their right hand. A continuous train of items paired with right-side tones followed by a left-side tone results in a boundary and creates a break in the temporal associations between items. **(C)** In the ABE, items presented concurrently with targets are enhanced. However, there is no evidence that target detection disrupts temporal context representations or that it influences the formation of inter-item associations. Image attributions: As in Figure 1, additionally, video stills reproduced with permission from Khena Swallow taken from her personal library.

If our account of the ABE is correct, then it may reflect how behaviorally relevant events (those that require a response) dynamically modulate attention and memory over time in naturalistic situations. However, research on how naturalistic events influence attention and memory has been conducted more or less in parallel to examinations of the ABE, with few attempts to integrate these literatures. In the next section, we address this gap in the literature. We start by describing research on event segmentation that converges on two broad effects on episodic memory: (1) the enhancement of information presented with an event boundary, and (2) the separation of representations of what happened before an event boundary from what happened next (Figure 3).

In naturalistic viewing, event segmentation is measured by asking participants to press a button to mark the boundaries between naturalistic units of activity as they watch a movie (e.g., of someone doing the dishes; Newtson, 1973). People tend to identify event boundaries when the situation changes in meaningful ways (i.e., when spatial configurations change; Schwan and Garsoffky, 2004; Zacks et al., 2010; Baker and Levin, 2015; Figure 3A) and the boundaries people identify track significant changes in brain activity and cognition during task-free viewing (e.g., Baldassano et al., 2017). More recently, laboratory tasks have examined event segmentation by creating context shifts (Figure 3B) that signal, for example, which task to perform on an item (DuBrow and Davachi, 2013, 2014, 2016), which hand to use to respond on that task (Clewett et al., 2020), or that increase reward prediction error (Rouhani et al., 2020). Despite using very different methods, research using naturalistic events and laboratory tasks both support the view that event representations are updated at event boundaries.

Both types of studies provide strong evidence that attention and memory are boosted at event boundaries, just as in the ABE. Attention to naturalistic movies increases (Faber et al., 2018; Kosie and Baldwin, 2019), encoding is enhanced (Ben-Yakov and Henson, 2018), and perceptual and conceptual information encountered at these times is better remembered than information encountered during nonboundary periods (Newtson and Engquist, 1976; Lassiter and Slaw, 1991; Swallow et al., 2009, 2011). Controlled laboratory tasks similarly find enhanced hippocampal activity at context changes (DuBrow and Davachi, 2016; Bulkin et al., 2020) and a memory advantage for items presented at, or soon after context changes during an encoding task (Heusser et al., 2018; Clewett et al., 2020; Rouhani et al., 2020).

Event segmentation plays a critical role in organizing episodic memory as well (Sargent et al., 2013). In naturalistic perception, segmentation influences the accessibility of information within versus across events (Radvansky and Copeland, 2006; Swallow et al., 2009, 2011; Kurby and Zacks, 2021), the quality of later memory (Kurby and Zacks, 2011), and estimates about their duration (Faber et al., 2018). Controlled laboratory tasks provide additional evidence that event segmentation organizes episodic memory: relative to objects presented in two different events, judgments about the temporal order of objects presented in the same event are enhanced (DuBrow and Davachi, 2013, 2014, 2016) and estimates of their temporal proximity to each other are decreased (Ezzyat and Davachi, 2014). Context changes during encoding also influence duration judgments (Brunec et al., 2017) and the order in which objects are later recalled (Heusser et al., 2018). These results thus demonstrate that boundaries influence how episodic memories are organized and subsequently remembered.

From this quick review, it is clear that the ABE is comparable to some effects of event boundaries on episodic memory, but it may not be comparable to others. The ABE is consistent with the boundary advantage because it shows that behaviorally relevant changes in task demands trigger a transient increase in attention and memory encoding (Figure 3C). It also parallels other aspects of the boundary advantage: the potential for incorporating information that is outside the current focus of goal-directed attention (Swallow et al., 2009, 2011; Yebra et al., 2019; Broitman and Swallow, 2020), better memory for which task was performed on which image (Heusser et al., 2018; Swallow and Atir, 2018; Turker and Swallow, 2019; Clewett et al., 2020; Mulligan et al., 2021), and the inclusion of features that distinguish category exemplars (Swallow et al., 2009; Sisk and Lee, 2021). While there is some evidence that the ABE also includes relational memory and spatial configurations (Swallow and Jiang, 2010; Leclercq et al., 2014; Turker and Swallow, 2019), we are not aware of similar effects having been examined in the segmentation literature.

Event segmentation also appears to impact memory in ways that have not yet been observed in the ABE. In contrast to segmentation, there is no evidence that responding to targets in the continuous dual-task encoding paradigm influences temporal context memory. The available data are not encouraging. In two studies, Mulligan et al. (2016, 2021) reported that measures that are sensitive to inter-item associative memory or when a word appeared are not influenced by target detection. Variability in attention over time also may have little effect on temporal context memory (Jayakumar et al., 2022). It is possible that the transient nature of responses in the continuous dual-task paradigm boosts episodic memory while having little effect on the formation of inter-item associations (cf. Heusser et al., 2018), especially in designs that present targets and distractors at equal rates (e.g., Swallow and Jiang, 2012; cf. McDaniel and Bugg, 2008). In contrast, event segmentation tasks maintain low-level perceptual cues or tasks over extended periods of time, creating new stable, temporally extended contexts in which items or actions can be associated (Grella et al., 2019; Rouhani et al., 2020).

These effects are consistent with the perspective highlighted in this review: that internal, stable states that generate efficient cognition are updated in response to mismatches between those states and the environment (Zacks et al., 2007; Friston, 2009; Brunec et al., 2018). However, they highlight important differences between paradigms that produce the ABE and those used to examine event segmentation. We propose that responding to targets in a continuous detection task boosts attention and encoding, just like event boundaries (Figure 3). However, responses in this task are also importantly different than responses in segmentation paradigms. In naturalistic perception, event boundaries require a response that results in an extended shift in the state of the system, promoting the formation of within event inter-item associations. In contrast, in the continuous dual-task paradigm, aligning the state of the system to the state of the environment requires a transient response (pressing a button, counting, temporarily withholding a button press). We suggest that this is the primary source of the difference between the ABE and the effects of event segmentation on episodic memory. However, it will be necessary for future research to test this possibility, as research on the ABE and event segmentation have tended to focus on different aspects of episodic memory.

### Boosting State Changes by Disrupting Top-Down Control

To better account for the effects of event boundaries on memory, we propose that phasic LC activity could contribute to memory by temporarily increasing cognitive flexibility under the right circumstances (dashed purple line in Figure 2). This is a speculative proposal, but it provides a more complete description of the impact of phasic LC activity on brain function. It also provides a straightforward basis for characterizing why responses to targets and event boundaries have common and uncommon effects on memory: whereas a transient response is needed to align the system after a target is detected, adapting to situational changes at event boundaries requires a larger and/or longer lasting shift in an animal’s internal state. As a result, context representations should shift more in response to event boundaries than to targets, and disruptions to the formation of inter-item associations in memory should be larger.

Several perspectives of phasic LC activity link it to this type of cognitive shift. Theoretical accounts of LC function suggest that it signals contextual volatility (Yu and Dayan, 2005; Parr and Friston, 2017) and facilitates the emergence of new functional states (Bouret and Sara, 2005). Consistent with these proposals, recent neuroimaging work finds evidence that LC activity could promote cognitive shifts by reducing the threshold for transitioning to a new state (Munn et al., 2021) and integrating processing across the brain (Shine et al., 2016). Similarly, the contributions of phasic LC activity to Go/No-Go task performance was captured in a computational model by a parameter that sped the decay of old control states, increasing the influence of more recent experiences (Sales et al., 2019). LC activity may even promote cognitive flexibility in the presence of cues that once predicted aversive events (Uematsu et al., 2017). Together, these findings suggest that phasic LC activation (probably in concert with other neuromodulatory systems) could promote cognitive flexibility.

In controlled laboratory tasks examining segmentation, increased cognitive flexibility might allow an animal to rapidly integrate new sources of information to adapt to changed contingencies between the environment (e.g., the stimuli that appear in an encoding task) and internal task sets (e.g., whether one should judge their size or whether they are likely to be found inside or outside). In naturalistic situations, increasing cognitive flexibility at event boundaries may facilitate the adoption of new event models and goals that better match the new situation. The contributions of phasic LC to shifts in HPC representations could also contribute to these shifts, segmenting episodic representations as they unfold (DuBrow and Davachi, 2016; Ben-Yakov and Henson, 2018; Grella et al., 2019; Clewett et al., 2020). LC mediated pattern separation could be one basis for this effect (Segal et al., 2012; Rouhani et al., 2020).

One implication of this idea is that, by increasing cognitive flexibility, phasic LC activation may transiently weaken (but not eliminate) goal-oriented attention during encoding. Though speculative, this possibility may account for observations that the ABE can incorporate information that is irrelevant to the ongoing task (see *“Goal-directed attention modulates the effects of temporal selection on encoding”*), while still allowing for interactive effects of temporal selection with the intention to memorize background items. If phasic LC activity weakens control states then top-down inhibition of task-irrelevant background items may decrease at the same time sensory gain increases. This account has similarities to explanations for the role of attention in task-irrelevant perceptual learning. Task-irrelevant perceptual learning occurs for stimuli paired with a target, but these effects are most reliable for stimuli that are presented below threshold, or outside focused attention, making them less likely to be inhibited by control mechanisms (Tsushima et al., 2008; Choi et al., 2009). It also overlaps with proposals suggesting that the attentional blink (the impaired ability to detect a target that occurs 200–500 ms after an earlier target), could reflect disruptions to control or selection mechanisms (Kawahara et al., 2006; Zivony and Lamy, 2021). In a similar way, disrupting control could also result in the inclusion of task-irrelevant information by the ABE. Finally, increased cognitive flexibility could explain why selecting stimuli for one task sometimes increases the likelihood that a prepared button press will be erroneously produced (Jiang and Swallow, 2014).

We emphasize, however, that weakening control states when a response is required does not mean that their influence is erased (cf. Sales et al., 2019). In the continuous dual-task that produces the ABE, instructions to memorize the background items should be maintained, even if a transient weakening increases the likelihood that irrelevant information will be caught up by temporal selection. Interactive effects of temporal selection with goal-directed attention (e.g., as in Mather et al., 2016) should therefore occur even if phasic LC activity briefly disrupts control. Indeed, higher rates of LC activity may be more likely to cause state transitions than a single phasic response on its own (Chandler, 2016). These considerations call for a careful evaluation of whether larger effects on memory would result from stronger or more arousing responses, as well as computational or cognitive modeling to capture the dynamics of the various effects of phasic LC on brain wide processing and how it is modulated by longer-lasting changes in arousal.

### Modular Circuitry and Function of the LC

Rapid advances in understanding the structure and function of the LC of the last 5–10 years (some of which was reviewed above) have led to the broad recognition that the LC may have a modular organization and the ability to shift between global and localized activity patterns (Poe et al., 2020). Rather than being a uniform nucleus that globally modulates neural activity, the LC may consist of multiple functional units that differ in their projection targets. These include separable projections to brain areas that could play an important role in generating responses to targets or changes in events: sensory and perceptual processing areas, hippocampal and limbic systems, and frontal control systems (Chandler, 2016; Poe et al., 2020).

The release of NE globally or at specific sites could lead to diverse effects on cognition. For example, in rats, separate cellular ensembles within the LC were found to have distinct and opposing effects on avoidance learning and extinction, *via* projections targeting prefrontal cortex and the amygdala, respectively (Uematsu et al., 2017). Single-unit recordings in both rats (Totah et al., 2018; Chandler et al., 2019) and monkeys (Usher et al., 1999; Joshi and Gold, 2022) demonstrate that levels of synchronous firing in the LC vary with cognitive state. Recent evidence further suggests that distinct LC ensembles produce distinct cognitive states in rats (Noei et al., 2022). Taken together, advances in characterizations of LC function and projection patterns describe a neuromodulatory system that is more sophisticated and precise than previously believed, and yet has an overarching purpose of facilitating changes of cognitive state as needed.

A natural question arising from this new perspective is whether the ABE reflects activity of specific ensembles projecting to specific brain regions, or whether it may result from more global signaling. The circumstances under which LC ensembles function jointly or independently (and are therefore more modular) are not yet well understood (Poe et al., 2020). Numerous differences between the conditions that cause segmentation or generate the ABE versus those under which modular versus global firing patterns in the LC have been examined make predictions difficult. However, because independence may be more likely with milder inputs into the LC, whereas global firing may occur with stronger input (Schwarz and Luo, 2015) it is possible that the ABE reflects more modular LC signaling, whereas context shifts in segmentation studies may be more likely to produce global LC signaling. The relatively new nature of these ideas demand caution, but also provide exciting new ways to think about the mechanisms by which attention and memory reflect neuromodulation by this and other systems.

## Conclusion

Attention and memory are coupled to changes in the external world. In this paper we suggest that this is because they are modulated by systems that stabilize internal representations of the world and update them when they no longer adequately reflect what is currently happening. A knock at the door, a partner’s call for help bringing the groceries in, or the sound of a cat knocking a plant off of a stand while one is reading all signal that the world has changed and so too must one’s model of it. In the perspective we have outlined here, for example, the sound of the pot crashing to the floor causes a mismatch between one’s internal representation of the situation (that the surroundings are amenable to reading) and the actual state of the world (a sudden noise suggests that something has broken). Because this mismatch could change how one should act in this situation, LC activity briefly increases, boosting sensory gain to facilitate the uptake of external information, individuating that moment from others, and causing a shift in event representations. At the same time, phasic LC activity could increase cognitive flexibility by promoting the decay of old states. Such flexibility would allow one to more rapidly adopt new, context appropriate states to then chase after the cat. Alternatively, if the crash one heard was just the cat jumping off the table, one could return to the book and the old state would persist.

In our view, the ABE is one manifestation of the mechanisms that tie attention and memory to changes in ongoing events. Just as in the cat example, we suggest that the ABE reflects the transient mobilization of effort to compensate for differences between internal states and the external world, and that this is mediated by the LC neuromodulatory system. Importantly, the effects of responses on memory are not limited to the paradigms that produce the ABE. Evidence from research on event segmentation using both naturalistic and controlled laboratory tasks shows that moments when events change are also moments that are more likely to be remembered. However, unlike in the ABE, changes in everyday events also create new contexts, leading to longer lasting, rather than transient, shifts in representations that guide cognition and behavior. In both cases, however, the critical factor regulating attention and memory is the need to respond to change.

## Author Contributions

KS, AB, ER, and HT contributed to the development of ideas presented in this paper. KS and AB outlined the paper. KS drafted the majority of the manuscript. All authors contributed to the article and approved the submitted version.

## Funding

The development of this manuscript was supported by the College of Arts and Sciences, Cornell University and the National Institutes of Health (National Institute on Aging grant F32 AG058479 to ER).

## Conflict of Interest

The authors declare that the research was conducted in the absence of any commercial or financial relationships that could be construed as a potential conflict of interest.

## Publisher’s Note

All claims expressed in this article are solely those of the authors and do not necessarily represent those of their affiliated organizations, or those of the publisher, the editors and the reviewers. Any product that may be evaluated in this article, or claim that may be made by its manufacturer, is not guaranteed or endorsed by the publisher.
